# *Gnathostoma spinigerum* Mitochondrial Genome Sequence: a Novel Gene Arrangement and its Phylogenetic Position within the Class Chromadorea

**DOI:** 10.1038/srep12691

**Published:** 2015-07-31

**Authors:** Guo-Hua Liu, Renfu Shao, Xian-Quan Cai, Wen-Wen Li, Xing-Quan Zhu

**Affiliations:** 1 State Key Laboratory of Veterinary Etiological Biology, Key Laboratory of Veterinary Parasitology of Gansu Province, Lanzhou Veterinary Research Institute, Chinese Academy of Agricultural Sciences, Lanzhou, Gansu Province 730046, P. R. China; 2 Genecology Research Centre, University of the Sunshine Coast, Queensland 4558, Australia; 3 Zhongshan Entry-Exit Inspection and Quarantine Bureau, Zhongshan, Guangdong Province 528403, P. R. China; 4 Department of Animal Experiment Center, The First Affiliated Hospital of Sun Yat-sen University, Guangzhou, Guangdong Province 510080, P. R. China

## Abstract

Human gnathostomiasis is an emerging food-borne parasitic disease caused by nematodes in the genus *Gnathostoma*. In spite of their significance as pathogens, these parasites remain poorly understood at the molecular level. In the present study, we sequenced the mitochondrial (mt) genome of *G. spinigerum*, which infects a range of definitive hosts including dogs, cats, tigers, leopards and humans. The mt genome of *G. spinigerum* is 14,079 bp in size and shows substantial changes in gene order compared to other nematodes studied to date. Phylogenetic analyses of mt genome sequences by Bayesian inference (BI) revealed that the infraorder Gnathostomatomorpha (represented by *G. spinigerum*) is closely related to the infraorder Ascaridomorpha. *G. spinigerum* is the first species from the infraorder Gnathostomatomorpha for which a complete mt genome has been sequenced. The new data will help understand the evolution, population genetics and systematics of this medically important group of parasites.

Human gnathostomiasis caused by *Gnathostoma* spp. is a highly endemic disease in some under-developed communities in Asia, particularly in China^1^. Recently, it has become an emerging disease among travelers from Europe and other continents who are coming into contact with endemic areas^2^. Nematodes of the genus *Gnathostoma* (Nematoda: Gnathostomatidae) are the etiological agents of human gnathostomiasis and may also infest dogs, cats, tigers and leopards^3^. There are 12 species in this genus with four species recorded in humans: *G. spinigerum*, commonly found in India, China, Japan and southeast Asia; *G. hispidum*, found in Europe, Asia and Australia; *G. doloresi*, found in southeast Asia; and *G. nipponicum*, found in Japan^3^. *G. spinigerum* is frequently reported in Asia as being responsible for human gnathostomiasis, but *G. hispidum*, *G. doloresi*, and *G. nipponicum* have also occasionally been reported^4^,^5^,^6^. Humans are infected when consuming raw or uncooked infected meats of intermediate hosts (fish) or other paratenic hosts (snake, pig and poultry). The majority of gnathostomiasis patients present with cutaneous lesions; involvement of eyes and central nervous system has also been sporadically reported^7^,^8^,^9^.

The genus *Gnathostoma* is in the infraorder Gnathostomatomorpha of the suborder Spirurina. There are four other infraorders in this suborder: Ascaridomorpha, Spiruromorpha, Rhigonematomorpha and Oxyuridomorpha^10^. The phylogenetic relationships among the infraorders of the Spirurina have been assessed using nuclear small subunit (SSU) rRNA (five infraorders) gene and mitochondrial (mt) gene/genome sequences (four infraorders) and there are inconsistencies between the nuclear phylogeny and the mt phylogeny^11^,^12^,^13^,^14^,^15^,^16^,^17^,^18^. In the phylogeny inferred from SSU rRNA gene sequences, the Ascaridomorpha is sister to the Rhigonematomorpha, and the Spiruromorpha is sister to the Ascaridomorpha + Rhigonematomorpha, the Oxyuridomorpha is sister to the Spiruromorpha + Ascaridomorpha + Rhigonematomorpha, and the Gnathostomatomorpha is sister to the Oxyuridomorpha + Spiruromorpha + Ascaridomorpha + Rhigonematomorpha^12^,^19^. In the mt gene/genome phylogeny, however, the Ascaridomorpha is sister to the Rhabditomorpha + Diplogasteromorpha in most analyses, and the Spiruromorpha is sister to the Rhabditomorpha + Diplogasteromorpha + Ascaridomorpha + Rhigonematomorpha + Panagrolaimomorpha + Tylenchomorpha + Oxyuridomorpha^15^,^16^,^17^. More recently, Kim *et al.*^20^ inferred the phylogeny with mt genome sequences and showed that the Rhigonematomorpha is sister to the Ascaridomorpha. Taxon sampling was limited in both the nuclear SSU rRNA and the mt gene/genome phylogenetic analysis; furthermore, no species from the Gnathostomatomorpha was included in any of these mt analyses.

Animal mt genomes are typically a circular DNA, 15–20 kb in size, containing 36–37 genes: 12–13 protein-coding genes (PCGs), 22 transfer RNAs (tRNA) and two ribosomal RNA (rRNA) genes^21^,^22^. Mt genome sequences are commonly used for phylogentic, population genetic and taxonomic investigations of animals^23^,^24^. To understand the phylogenetic relationship of the infraorder Gnathostomatomorpha with other infraorders of the class Chromadorea, we sequenced the mt genome of *G. spinigerum*.

## Results and Discussion

### General features of the mt genome of *G. spinigerum*

**Figure 1:**
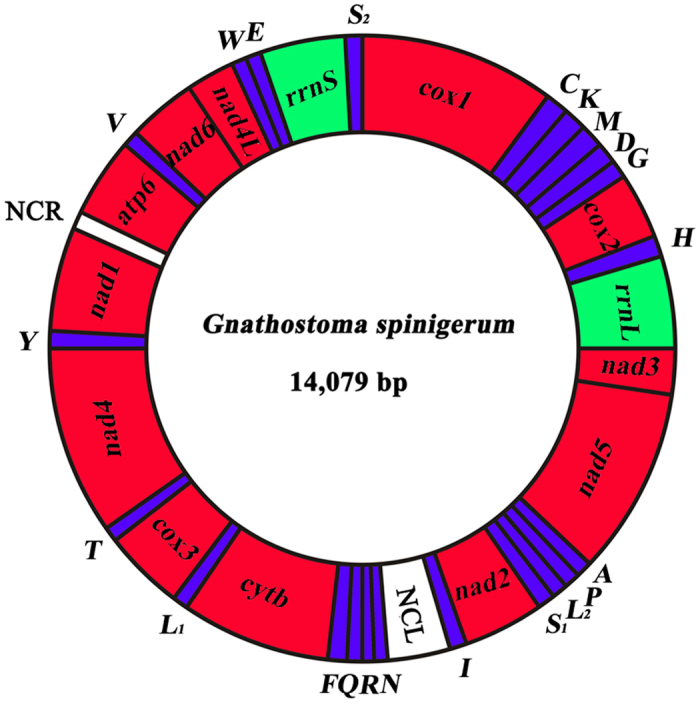
The mitochondrial genome of *Gnathostoma spinigerum.* All genes are on the same DNA strand and are transcribed clockwise. Protein-coding and rRNA genes are indicated with the standard nomenclature. tRNA genes are indicated with the one-letter code of their corresponding amino acids. There are two tRNA genes for leucine: L_1_ for codons CUN and L_2_ for UUR; and two tRNA genes for serine: S_1_ for codons AGN and S_2_ for UCN. “NCL” refers to the large non-coding region. “NCR” refers to a small non-coding region.

**Figure 2:**
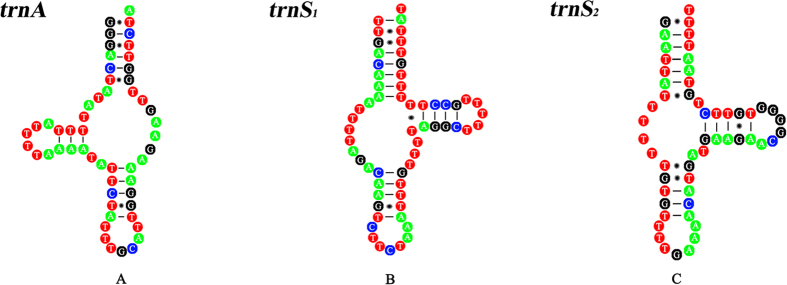
The predicted secondary structures of representing tRNAs of the *Gnathostoma spinigerum* mitochondrial DNA determined in this study.

The complete mt genome of *G. spinigerum* (GenBank accession no. KP410547) was 14,079 bp in size (Fig. 1). This genome contains 12 PCGs (*cox1-3*, *nad1-6*, *nad4L*, *atp6* and *cytb*), 22 tRNA genes, two rRNA genes (*rrnL* and *rrnS*) and two non-coding (AT-rich) regions. All genes are transcribed in the same direction. As in most other nematodes of the class Chromadorea, *atp8* gene is not present in the mt genome of *G. spinigerum* (Table 1). The mt genome sequence of *G. spinigerum* is biased toward A and T (71.1%), similar to that of other nematodes in the suborder Spirurina^25^,^26^,^27^,^28^. This nucleotide composition of the 12 PCGs of *G. spinigerum* was strongly skewed away from A, in favour of T (AT skew between −0.58 and –0.27), and the GC skew was between 0.41 and 0.77 (Table 2). Codons composed of A and T were more frequently used in PCGs, reflecting the high A + T content in the mt genome of *G. spinigerum*. The most frequently used amino acid was TTT (Phe; 13.14%), followed by TTG (Leu; 8.79%), ATT (IIe; 5.96%) and GTT (Val; 5.46%) (Table 3). ATA, TTG and ATT were used as initiation codons and TAA and TAG as termination codons; incomplete termination codons (T or TA) were also identified (Table 1), which is consistent with the arrangement in the mt genomes of other nematodes^29^,^30^,^31^. Twenty-two tRNA genes were identified in the mt genome of *G. spinigerum*, which range from 54 to 68 bp in size. The secondary structures inferred for the 22 tRNAs of *G. spinigerum* are similar to those of other nematodes^29^,^30^,^31^ (Fig. 2). *rrnL* is located between *trnH* and *nad3* in the *G. spinigerum* mt genome; *rrnS* is between *trnE* and *trnS*_*2*_ (Table 1). The two non-coding regions in the mt genome of *G. spinigerum* were located between *trnI* and *trnN* (designated NCL, 750 bp), and between *nad1* and *atp6* (designated NCR, 77 bp) respectively. No repetitive sequences were detected in the non-coding regions of *G. spinigerum*, as in other Spirurina nematodes^26^,^27^.

### Gene arrangement in the mt genome of *G. spinigerum*

The mt genome of *G. spinigerum* shows a different gene arrangement pattern from the other 29 patterns seen in nematodes revealed by previous studies (pattern GA26 hereafter; see^17^ for GA1-25; GA27 for *Rhigonema thysanophora*^20^; GA28-30 for *Meloidogyne chitwoodi*, *M. graminicola* and *M. incognita*, respectively^32^,^33^). In this study, we compared the 30 gene arrangement patterns in nematodes, and found that the gene order for all of the protein-coding and rRNA genes were principally conserved, but tRNA genes/clusters were not conserved in nematodes. Furthermore, mt gene arrangement events among the 30 patterns observed in nematodes were analyzed with CREx^34^; numerous transposition and tandem-duplication-random-loss (TDRL) events could be inferred. Our results support the view that the evolution of mt gene order in nematodes was mostly driven by transposition and TDRL^14^; inversion and reverse-transposition played a minor role relatively.

**Figure 3:**
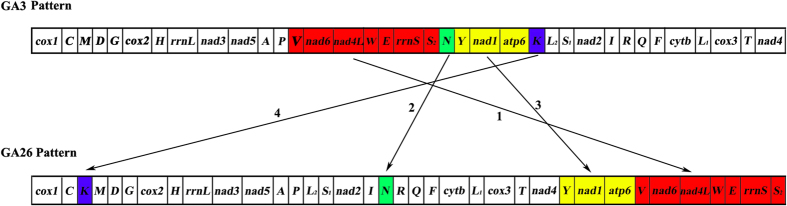
Rearrangement of mitochondrial genes in *Gnathostoma spinigerum* (pattern GA26) relative to the most common pattern of mitochondrial gene arrangement observed in nematodes (GA3).

Compared to the most common pattern of mt gene arrangement seen in nematodes (i.e. GA3)^17^, a block of 12 genes (from *trnV* to *trnK*, Fig. 3) in *G. spinigerum* has been broken and moved to four locations. Seven genes (*trnV*, *nad6*, *nad4L*, *trnW*, *trnE*, *rrnS* and *trnS*_*2*_) between *trnP* and *trnN* in GA3 pattern, was found between *apt6* and *cox1* in GA26 pattern in *G. spinigerum*. *trnN* was located between *trnS*_*2*_ and *trnY* in GA3 pattern but was between *trnI* and *trnR* in GA26 pattern. Three genes (*trnY*, *nad1* and *atp6*) between *trnN* and *trnK* in GA3 pattern were located between *nad4* and *trnV* in GA26 pattern. Additionally, *trnK*, which was between *atp6* and *trnL*_*2*_ in GA3 pattern, was located between *trnC* and *trnM* in GA26 pattern. CREx analysis modeled that one transposition and two TDRL events were required to convert GA3 to GA26. Two rearrangement events involved PCGs while the other two events involved only tRNA genes.

### Phylogenetic analyses

**Figure 4:**
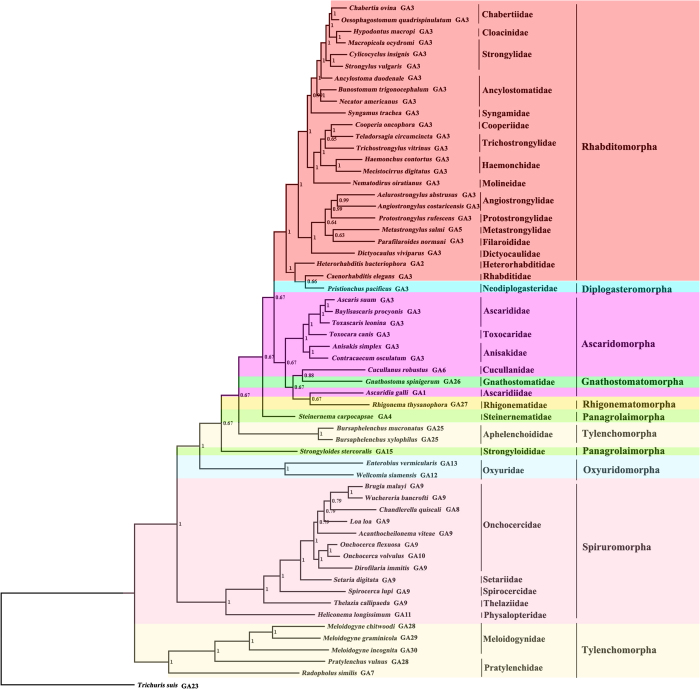
Phylogenetic relationships among 58 species of Chromadorea nematodes inferred from Bayesian inference of deduced amino acid sequences of 12 mitochondrial protein-coding genes. *Trichuris suis* (GenBank accession number GU070737) was used as the outgroup. Bayesian posterior probabilities (Bpp) values were indicated at nodes.

We inferred the phylogenetic relationship between *G. spinigerum* and other 57 species of Chromadorea nematodes with concatenated amino acid sequences of the 12 mt PCGs (Fig. 4). Phylogenies of the Chromadorea nematodes were inferred with mt genome sequences in previous studies^14^,^15^,^16^,^17^,^18^; however, several major lineages including the infraorder Gnathostomatomorpha were not represented.

Our Bayesian analysis showed that *G. spinigerum* was most closely related to *Cucullanus robustus* with moderate support [Bayesian posterior probabilities (Bpp) = 0.88, Fig. 4]. Our maximum likelihood (ML) and maximum parsimony (MP) analyses also recovered this relationship but the bootstrapping frequency (Bf) was weak (not shown). This grouping was inconsistent with those from morphological and molecular studies^12^,^19^,^35^,^36^,^37^. The infraorder Ascaridomorpha was monophyletic in the present study (Bpp = 0.67, Fig. 4). The two species from the two families, Cucullanidae and Ascaridiidae, of the infraorder Ascaridomorpha were more closely related to *G. spinigerum* (infraorder Gnathostomatomorpha) and *R. thysanophora* (infraorder Rhigonematomorpha) than they were to other six species from the infraorder Ascaridomorpha. *R. thysanophora* was most closely related to *Ascaridia galli* (Bpp = 0.67). A recent study based on mt genome sequences also indicated that *R. thysanophora* was most closely related to an *Ascaridia* species^20^. Using nuclear SSU rRNA gene sequences, Meldal *et al.*^12^ showed that the infraorder Ascaridomorpha was sister to the group that included the infraorders Ascaridomorpha, Rhabditomorpha, Spiruromorpha, Oxyuridomorpha, and Gnathostomatomorpha. These controversial results surrounding phylogenetic placement of members in Spirurina may reflect the different evolutionary rates of the nuclear and mt genomes^38^,^39^.

Our analysis also showed that the infraorder Rhabditomorpha was paraphyletic with respect to the Diplogasteromorpha. Two species from the families Rhabditidae and Heterorhabditidae of the Rhabditomorpha were more closely related to *Pristionchus pacificus* (Neodiplogasteridae) than they were to the other 22 species from the Rhabditomorpha. The close relationship between the species of the families Rhabditidae and Heterorhabditidae and *P. pacificus* was strongly supported in BI (Bpp = 1, Fig. 4). The results were consistent with that of a previous study using nuclear SSU rRNA gene dataset^11^. The Oxyuridomorpha (2 species) and the Spiruromorpha (12 species) were both monophyletic with strong support in the present analysis (Bpp = 1, Fig. 4). The nine species of the suborder Tylenchina included in this study were from two infraorders: Panagrolaimorpha (2 species), and Tylenchomorpha (7 species). Both of these infraorders were paraphyletic in the present analysis (Bpp ≥ 0.67, Fig. 4). The Oxyuridomorpha and the Spiruromorpha were both monophyletic with strong support in the current analyses (Bpp = 1 for Oxyuridomorpha and Bpp ≥ 0.79 for Spiruromorpha, Fig. 4).

For decades, there have been controversies surrounding the systematics of the suborder Spirurina (infraorders Ascaridomorpha, Spiruromorpha, Rhigonematomorpha, Gnathostomatomorpha and Oxyuridomorpha)^10^. Given the demonstrated utility of mt datasets, there is now an opportunity to test the phylogenetic relationships of a wide range of Spirurina nematodes using expanded mt datasets. Analyses of mt genome sequences in the current study and several previous studies^14^,^15^,^16^,^17^,^18^ have provided insights into the phylogenetic relationships among major lineages of the Spirurina nematodes. However, some lineages of the suborder Spirurina are underrepresented or not represented in these analyses. So, more mt genome data from the suborder Spirurina would be required in future analyses to understand the phylogeny of the suborder Spirurina.

In summary, this is the first determination of a complete mt genome of a parasite belonging to the infraorder Gnathostomatomorpha. Although the length, gene and AT content are similar to other nematode mt genomes, the mt genome of *G. spinigerum* exhibits some interesting features. The gene order of *G. spinigerum* is distinct from that of other nematodes. Phylogenetic analysis shows that *G. spinigerum* was most closely related to *Cucullanus robustus* with moderate support, which is inconsistent with that from morphological and molecular studies. Our results provided insights into the phylogenetic relationships among several major lineages of nematodes.

## Methods

### Ethics statement

Specimens of *G. spinigerum* were collected from an Asian swamp eel, in accordance with the animal ethics procedures and guidelines China. All experimental protocols were approved by the Animal Ethics Committee of Lanzhou Veterinary Research Institute, Chinese Academy of Agricultural Sciences.

### Collection of *G. spinigerum* and DNA isolation

Larval specimens of *G. spinigerum* were collected from an infected Asian swamp eel, *Monopterus albus*, imported from Indonesia, and were identified to species morphologically^40^. The specimens were fixed in ethanol and stored at –20 °C until use. Total genomic DNA was isolated from individual worms using small-scale sodium dodecyl-sulphate (SDS)/proteinase K digestion and spin-column purification (Wizard® SV Genomic DNA Purification System, Promega). The identity of *G. spinigerum* specimens (coded GS5) was also verified by sequencing regions of *ITS-2* and *cox1* genes^41^; both regions had 100% similarity with those of *G. spinigerum* from Thailand and Indonesia (GenBank accession Nos. AB181155 and JN408304).

### Long-PCR amplification and sequencing

Fragments of *cox1* and *nad1* genes of *G. spinigerum* were amplified by PCR with primer pairs JB3-JB4.5^42^ and JB11-JB12^43^ (Table 4). After we obtained partial *cox1* and *nad1* sequences for *G. spinigerum*, we designed specific primers from these fragments for long PCR amplification. The complete mt genome of *G. spinigerum* (coded GS5) was amplified by long-PCR as two segments (~10 kb and ~4 kb) using genomic DNA extracted from a single specimen; the gaps between the two segments were filled by the short *cox1* and *nad1* fragments amplified initially. PCR was conducted in 25 μl using 2 mM MgCl_2_, 0.2 mM each of dNTPs, 2.5 μl 10 × Taq buffer, 2.5 μM of each primer and 0.5 μl LA *Taq* DNA polymerase (5 U/μl, Takara) in a thermocycler (Biometra). The cycling conditions were: 92 °C for 2 min (initial denaturation), then 92 °C for 10 s (denaturation), 56 °C (10 kb) or 54 °C (4 kb) for 30 s (annealing) and 60 °C for 10 min (extension) for 10 cycles, followed by 92 °C for 10 s, 56 °C (~10 kb) or 54 °C (~4 kb) for 30 s (annealing), and 60 °C for 10 min for 20 cycles, with a cycle elongation of 10 s for each cycle and a final extension at 60 °C for 10 min. PCR products were sequenced at Sangon Company (Shanghai, China) using a primer walking strategy^44^.

### Sequence analyses

Sequences obtained from the PCR amplicons of *G. spinigerum* were assembled manually and aligned with the mt genome sequences of roundworm and pinworm (GenBank accession numbers: NC_016128 and NC_011300) using the program MAFFT 7.122^45^ to identify gene boundaries. The sequence of each protein-coding gene was translated into amino acid sequence using the invertebrate mt genetic code in MEGA 5^46^; the amino acid sequences were aligned using default settings. tRNAscan-SE^47^ and ARWEN^48^ were used to identify all of the tRNA genes except *trnS*_*2*_, which was identified manually by sequence comparison with *trnS*_*2*_ of other nematodes reported previously^49^. The two rRNA genes were identified by BLAST searches and were verified by sequence comparison with these two genes of other nematodes reported previously^14^. Tandem repeats in the non-coding regions were found using Tandem Repeat Finder program (http://tandem. bu.edu/trf/trf.html)^50^. The rearrangement events in the mt genomes were modelled with CREx (http://pacosy.informatik.uni-leipzig.de/crex)^34^.

### Phylogenetic analyses

We combined the mt genome sequence of *G. spinigerum* with those of selected 57 other Chromadorea nematodes (Table S1) retrieved from the GenBank for phylogenetic analysis; *Trichuris suis* (GenBank accession number GU070737) was used as the outgroup^51^. Amino acid sequences inferred from the sequences of 12 mt PCGs were aligned individually first using MAFFT 7.122 and were then concatenated to form a single dataset; ambiguously aligned regions were excluded using Gblocks 0.91b (doc)^52^ with the default parameters (allow smaller final blocks, allow gap positions within the final blocks and allow less strict flanking positions). Phylogenetic analyses were conducted using Bayesian inference (BI). The MtArt + I + G + F model of amino acid evolution was selected as the most suitable model of evolution by ProtTest 2.4^53^ based on the Akaike information criterion (AIC). As MtArt model is not implemented in the current version of MrBayes, an alternative model, CpREV, was used in BI and four chains (three heated and one cold) were run simultaneously for the Monte Carlo Markov Chain. Two independent runs for 2,000,000 metropolis-coupled MCMC generations, sampling a tree every 100 generations in MrBayes 3.1.1^54^; the first 5,000 trees represented burn-in and the remaining trees were used to calculate Bpp. Bayesian analysis was run until the potential scale reduction factor approached 1 and the average standard deviation of split frequencies was less than 0.01. All sites were coded as unordered and equally weighted characters. The topology was reconstructed using the 50% majority rule and the support values were assessed by 1000 bootstrap replicates. Phylograms were drawn using the program FigTree v.1.4 (http://tree.bio.ed.ac.uk/software/figtree).

## Additional Information

**How to cite this article**: Liu, G.-H. *et al.* *Gnathostoma spinigerum* Mitochondrial Genome Sequence: a Novel Gene Arrangement and its Phylogenetic Position within the Class Chromadorea. *Sci. Rep.* **5**, 12691; doi: 10.1038/srep12691 (2015).

## Supplementary Material

Supplementary Information

## Figures and Tables

**Table 1:** The organization of the mitochondrial genome of *Gnathostoma spinigerum.*

Genes	Positions	Lengths (bp)	Start codons	Stop codons	Anticodons
*cox1*	1–1572	1572	ATA	TAG	
tRNA-Cys (C)	1573–1629	57			GCA
tRNA-Lys (K)	1629–1691	63			TTT
tRNA-Met (M)	1699–1752	53			CAT
tRNA-Asp (D)	1753–1808	56			GTC
tRNA-Gly (G)	1813–1868	56			TCC
*cox2*	1869–2556	688	TTG	T	
tRNA-His (H)	2557–2612	56			GTG
*rrnL*	2613–3554	942			
*nad3*	3555–3890	336	TTG	TAA	
*nad5*	3894–5478	1585	TTG	T	
tRNA-Ala (A)	5479–5533	55			TGC
tRNA-Pro (P)	5535–5592	58			TGG
tRNA-Leu ^UUR^ (L_2_)	5598–5652	55			TAA
tRNA-Ser ^AGN^ (S1)	5651–5710	60			TCT
*nad2*	5721–6553	833	ATA	TA	
tRNA-Ile (I)	6554–6609	56			GAT
Non-coding region	6610–7359	750			
tRNA-Asn (N)	7360–7416	57			GTT
tRNA-Arg (R)	7448–7504	57			TCG
tRNA-Gln (Q)	7504–7557	54			TTG
tRNA-Phe (F)	7557–7624	68			GAA
*cytb*	7648–8721	1074	ATA	TAG	
tRNA-Leu ^CUN^ (L1)	8722–8776	55			TAG
*cox3*	8777–9544	768	TTG	TAG	
tRNA-Thr (T)	9547–9601	55			TGT
*nad4*	9602–10826	1225	TTG	T	
tRNA-Tyr (Y)	10827–10880	54			GTA
*nad1* Non-coding region	10881–11753 11754–11830	873 77	TTG	TAA	
*atp6*	11831–12409	579	ATT	TAG	
tRNA-Val (V)	12463–12517	55			TAC
*nad6*	12518–12955	438	TTG	TAG	
*nad4L*	12963–13190	228	TTG	TAG	
tRNA-Trp (W)	13191–13247	57			TCA
tRNA-Glu (E)	13255–13311	57			TTC
*rrnS*	13312–13985	674			
tRNA-Ser ^UCN^ (S2)	13986–14039	54			TGA

**Table 2:** Nucleotide composition and skews of the *Gnathostoma spinigerum* mitochondrial protein-coding genes.

Gene	Proportion of nucleotides				A + T (%)	AT Skew	GC Skew
	A	T	G	C			
*cox1*	20.6	46.4	23.4	9.7	67.0	−0.39	0.41
*cox2*	22.7	45.9	24.1	7.3	68.6	−0.34	0.54
*cox3*	19.8	50.9	21.2	8.1	70.7	−0.44	0.45
*nad1*	21.2	47.8	22.2	8.8	69.0	−0.39	0.43
*nad2*	25.0	51.0	17.6	6.4	76.0	−0.34	0.47
*nad3*	21.1	52.7	23.2	3.0	73.8	−0.43	0.77
*nad4*	20.2	51.2	21.1	7.5	71.4	−0.43	0.48
*nad4L*	15.8	57.0	22.4	4.8	72.8	−0.57	0.65
*nad5*	21.0	50.1	22.3	6.6	71.1	−0.41	0.54
*nad6*	15.3	57.1	22.4	5.3	72.4	−0.58	0.62
*cytb*	20.8	49.2	21.9	8.2	70.0	−0.41	0.46
*atp6*	25.9	44.7	22.1	7.3	70.6	−0.27	0.50

**Table 3:** Codon usage of *Gnathostoma spinigerum* mitochondrial protein-coding genes.

Amino acid	Codon	Number	Frequency (%)	Amino acid	Codon	Number	Frequency (%)
Phe	TTT	447	13.14	Met	ATA	92	2.70
Phe	TTC	13	0.38	Met	ATG	114	3.35
Leu	TTA	183	5.38	Thr	ACT	78	2.29
Leu	TTG	299	8.79	Thr	ACC	2	0.05
Ser	TCT	114	3.35	Thr	ACA	7	0.20
Ser	TCC	6	0.17	Thr	ACG	11	0.32
Ser	TCA	16	0.47	Asn	AAT	92	2.70
Ser	TCG	9	0.26	Asn	AAC	4	0.11
Tyr	TAT	177	5.20	Lys	AAA	43	1.26
Tyr	TAC	4	0.11	Lys	AAG	48	1.41
Stop	TAA	2	0.05	Ser	AGT	98	2.88
Stop	TAG	6	0.17	Ser	AGC	7	0.20
Cys	TGT	59	1.73	Ser	AGA	60	1.76
Cys	TGC	2	0.05	Ser	AGG	57	1.67
Trp	TGA	27	0.79	Val	GTT	186	5.46
Trp	TGG	46	1.35	Val	GTC	9	0.26
Leu	CTT	32	0.94	Val	GTA	62	1.82
Leu	CTC	1	0.02	Val	GTG	80	2.35
Leu	CTA	7	0.20	Ala	GCT	81	2.38
Leu	CTG	18	0.52	Ala	GCC	5	0.14
Pro	CCT	66	1.94	Ala	GCA	13	0.38
Pro	CCC	1	0.02	Ala	GCG	5	0.14
Pro	CCA	3	0.08	Asp	GAT	74	2.17
Pro	CCG	8	0.23	Asp	GAC	4	0.11
His	CAT	50	1.47	Glu	GAA	37	1.08
His	CAC	3	0.08	Glu	GAG	48	1.41
Gln	CAA	16	0.47	Gly	GGT	103	3.02
Gln	CAG	23	0.67	Gly	GGC	9	0.26
Arg	CGT	23	0.67	Gly	GGA	29	0.85
Arg	CGC	3	0.08	Gly	GGG	63	1.85
Arg	CGA	3	0.08	IIe	ATT	203	5.96
Arg	CGG	5	0.14	IIe	ATC	5	0.14

**Table 4:** Sequences of primers used to amplify PCR fragments from *Gnathostoma spinigerum.*

Name of primer	Sequence (5’ to 3’)	Reference
Short PCR		
JB3	TTTTTTGGGCATCCTGAGGTTTAT	42
JB4.5	TAAAGAAAGAACATAATGAAAATG	
JB11	AGATTCGTAAGGGGCCTAATA	43
JB12	ACCACTAACTAATTCACTTTC	
Long PCR		
GSCF	GGTTTTCGTATGATGTTTTCTCCTT	This study
GSNR	CCACCATTCCATACTTAGACTTCCT	
GSNF	CCTGGAGTCGCTTTTGTAACTATGT	This study
GSCR	GTAGCCAACCATCTAAAAACCTTCA	
