# Supplementary material for: Gnathostoma spinigerum Mitochondrial Genome Sequence: a Novel Gene Arrangement and its Phylogenetic Position within the Class Chromadorea
Source: Sci Rep. 2015 Jul 31;5:12691. doi: 10.1038/srep12691 (PMC4521153; doi:10.1038/srep12691)
Supplement: Supplementary Information [file srep12691-s1.docx]

***Gnathostoma spinigerum* M****itochondrial Genome Sequence: a Novel Gene Arrangement and its Phylogenetic Position within** **the Class Chromadorea**

**Guo-Hua Liu^1^, Renfu Shao^2^, Xian-Quan Cai^1,3^, Wen-Wen Li^4^ & Xing-Quan Zhu^1*^**

**^1^**State Key Laboratory of Veterinary Etiological Biology, Key Laboratory of Veterinary Parasitology of Gansu Province, Lanzhou Veterinary Research Institute, Chinese Academy of Agricultural Sciences, Lanzhou, Gansu Province 730046, P. R. China, **^2^**Genecology Research Centre, University of the Sunshine Coast, Queensland 4558, Australia, **^3^**Zhongshan Entry-Exit Inspection and Quarantine Bureau, Zhongshan, Guangdong Province 528403, P. R. China, **^4^**Department of Animal Experiment Center, The First Affiliated Hospital of Sun Yat-sen University, Guangzhou, Guangdong Province 510080, P. R. China

__________________________________________________________________

Correspondence and requests for materials should be addressed to X.-Q.Z. (xingquanzhu1@hotmail.com)

**Table S1 Selected mitochondrial genome sequences of Chromadorea nematodes used for phylogenetic analysis in the present study.** ***Gnathostoma spinigerum* is shown in bold*.***

| Species | Length (bp) | GenBank accession numbers |
| --- | --- | --- |
| *Acanthocheilonema viteae*  *Aelurostrongylus abstrusus*  *Ancylostoma duodenale*  *Angiostrongylus costaricensis*  *Anisakis simplex*  *Ascaridia galli*  *Ascaris suum*  [*Baylisascaris procyonis*](http://www.ncbi.nlm.nih.gov/Taxonomy/Browser/wwwtax.cgi?lvl=0&id=6259)  *Bursaphelenchus mucronatus*  *Bursaphelenchus xylophilus*  *Brugia malayi*  *Bunostomum trigonocephalum*  *Caenorhabditis elegans*  *Chabertia ovina*  *Chandlerella quiscali*  *Contracaecum osculatum*  *Cooperia oncophora*  *Cylicocyclus insignis*  *Cucullanus robustus*  *Dictyocaulus viviparous*  *Dirofilaria immitis*  *Enterobius vermicularis*  ***Gnathostoma spinigerum***  *Haemonchus contortus*  *Heliconema longissimum*  *Heterorhabditis bacteriophora*  *Hypodontus macropi*  *Loa loa*  *Macropicola ocydromi*  *Mecistocirrus digitatus*  *Metastrongylus salmi*  *Meloidogyne chitwoodi*  *Meloidogyne graminicola*  *Meloidogyne incognita*  *Necator americanus*  *Nematodirus oiratianus*  *Oesophagostomum quadrispinulatum*  *Onchocerca flexuosa*  *Onchocerca volvulus*  *Parafilaroides normani*  *Pratylenchus vulnus*  *Pristionchus pacificus*  *Protostrongylus rufescens*  *Radopholus similis*  *Rhigonema thysanophora*  *Setaria digitata*  *Spirocerca lupi*  *Strongylus vulgaris*  *Steinernema carpocapsae*  *Strongyloides stercoralis*  *Syngamus trachea*  *Teladorsagia circumcincta*  *Thelazia callipaeda*  *Toxascaris leonina*  *Toxocara canis*  *Trichostrongylus vitrinus*  [*Wellcomia siamensis*](http://www.ncbi.nlm.nih.gov/Taxonomy/Browser/wwwtax.cgi?lvl=0&id=435744)  *Wuchereria bancrofti* | 13724  13913  13721  13585  13916  13977  14284  14781  14583  14778  13657  13764  13794  13682  13757  13823  13636  13828  13972  13310  13814  14010  **14079**  14055  13610  18128  13634  13590  13659  15221  13778  18201  20030  17662  13605  13765  13681  13672  13747  13414  21656  15954  13619  16791  15015  13839  13780  14301  13925  13758  14647  14066  13668  14310  14322  13800  14128  13635 | NC_016197  [NC_019571](http://www.ncbi.nlm.nih.gov/nuccore/426580925)  [NC_003415](http://www.ncbi.nlm.nih.gov/nuccore/19073878)  [NC_013067](http://www.ncbi.nlm.nih.gov/nuccore/255506291)  [NC_007934](http://www.ncbi.nlm.nih.gov/nuccore/91176175)  [NC_021642](http://www.ncbi.nlm.nih.gov/nuccore/521258298)  NC_001327  [NC_016200](http://www.ncbi.nlm.nih.gov/nuccore/357018143)  NC_021120  NC_023208  [NC_004298](http://www.ncbi.nlm.nih.gov/nuccore/23395805)  [NC_019803](http://www.ncbi.nlm.nih.gov/nuccore/429222004)  [NC_001328](http://www.ncbi.nlm.nih.gov/nuccore/5834884)  [NC_013831](http://www.ncbi.nlm.nih.gov/nuccore/288903409)  [NC_014486](http://www.ncbi.nlm.nih.gov/nuccore/306960077)  NC_024037  [NC_004806](http://www.ncbi.nlm.nih.gov/nuccore/30725254)  [NC_013808](http://www.ncbi.nlm.nih.gov/nuccore/288900705)  [NC_016128](http://www.ncbi.nlm.nih.gov/nuccore/353526742)  [NC_019810](http://www.ncbi.nlm.nih.gov/nuccore/429222094)  [NC_005305](http://www.ncbi.nlm.nih.gov/nuccore/40548800)  [NC_011300](http://www.ncbi.nlm.nih.gov/nuccore/207266404)  **KP410547**  [NC_010383](http://www.ncbi.nlm.nih.gov/nuccore/195972419)  [NC_016127](http://www.ncbi.nlm.nih.gov/nuccore/353526729)  [NC_008534](http://www.ncbi.nlm.nih.gov/nuccore/116510830)  NC_023098  [NC_016199](http://www.ncbi.nlm.nih.gov/nuccore/357018130)  NC_023099  [NC_013848](http://www.ncbi.nlm.nih.gov/nuccore/288904190)  [NC_013815](http://www.ncbi.nlm.nih.gov/nuccore/288903217)  NC_024096  NC_024275  NC_024097  [NC_003416](http://www.ncbi.nlm.nih.gov/nuccore/34582511)  NC_024639  [NC_014181](http://www.ncbi.nlm.nih.gov/nuccore/296940307)  [NC_016172](http://www.ncbi.nlm.nih.gov/nuccore/357017812)  AF015193  KJ801815  NC_020434  NC_015245  NC_023262  [NC_013253](http://www.ncbi.nlm.nih.gov/nuccore/258649588)  NC_024020  [NC_014282](http://www.ncbi.nlm.nih.gov/nuccore/299828992)  KC305876  [NC_013818](http://www.ncbi.nlm.nih.gov/nuccore/294368067)  [NC_005941](http://www.ncbi.nlm.nih.gov/nuccore/49146518)  NC_005143  [NC_013821](http://www.ncbi.nlm.nih.gov/nuccore/288903284)  [NC_013827](http://www.ncbi.nlm.nih.gov/nuccore/288903352)  JX069968  NC_023504  [NC_010690](http://www.ncbi.nlm.nih.gov/nuccore/188011112)  NC_013807  [GQ332427](http://www.ncbi.nlm.nih.gov/nuccore/GQ332427)  JN367461 |
